# Vitamin A Derivatives as Treatment Options for Retinal Degenerative Diseases

**DOI:** 10.3390/nu5072646

**Published:** 2013-07-12

**Authors:** Lindsay Perusek, Tadao Maeda

**Affiliations:** Department of Ophthalmology & Visual Sciences, School of Medicine, Case Western Reserve University, Cleveland, OH 44106-4965, USA; E-Mail: lxp106@case.edu

**Keywords:** vitamin A, all-*trans*-retinol, 11-*cis*-retinal, retina, visual cycle, carotenoids, 9-*cis*-retinyl acetate, retinal pigmented epithelium, photoreceptor, Leber congenital amaurosis

## Abstract

The visual cycle is a sequential enzymatic reaction for vitamin A, all-*trans*-retinol, occurring in the outer layer of the human retina and is essential for the maintenance of vision. The central source of retinol is derived from dietary intake of both retinol and pro-vitamin A carotenoids. A series of enzymatic reactions, located in both the photoreceptor outer segment and the retinal pigment epithelium, transform retinol into the visual chromophore 11-*cis*-retinal, regenerating visual pigments. Retina specific proteins carry out the majority of the visual cycle, and any significant interruption in this sequence of reactions is capable of causing varying degrees of blindness. Among these important proteins are Lecithin:retinol acyltransferase (LRAT) and retinal pigment epithelium-specific 65-kDa protein (RPE65) known to be responsible for esterification of retinol to all-*trans*-retinyl esters and isomerization of these esters to 11-*cis*-retinal, respectively. Deleterious mutations in these genes are identified in human retinal diseases that cause blindness, such as Leber congenital amaurosis (LCA) and retinitis pigmentosa (RP). Herein, we discuss the pathology of 11-*cis*-retinal deficiency caused by these mutations in both animal disease models and human patients. We also review novel therapeutic strategies employing artificial visual chromophore 9-*cis*-retinoids which have been employed in clinical trials involving LCA patients.

## 1. Introduction

Vitamin A is an essential vitamin for vertebrates and therefore must be obtained from dietary sources. Intake of adequate vitamin A is required in adults to maintain immune system integrity, vision, and the regulation of gene transcription, while in embryo development it is required for organogenesis, tissue differentiation and hematopoiesis [1,2,3]. Total vitamin A intake consists of many dietary forms including retinyl esters, β-carotene and free retinol. The various ingested forms of vitamin A are then processed, stored in the liver and can be released into the systemic circulation upon demand.

The various functions of vitamin A are carried out by several metabolically active derivatives including 11-*cis*-retinal and all-*trans*-retinoic acid, which are required for vision and transcriptional gene regulation respectively [4,5]. Enzymatic activity and gene regulation within many tissues is dependent upon a consistent supply of vitamin A, therefore tissues such as the eye, possess cyclic series of reactions to regenerate biologically active vitamin A such as a visual chromophore 11-*cis*-retinal. The efficient renewal of vitamin A is termed the visual cycle, and is indispensable for normal vision in humans. Genetic mutations which result in faulty enzymes required for the eye specific processing of vitamin A may cause early onset retinal degeneration due to the lack of 11-*cis*-retinal chromophore and ultimately can lead to complete loss of vision. Retinoid therapies utilize specific isomers of vitamin A and have proven to be effective at alleviating progressive tissue damage in animal models of retinal degeneration. Retinoid treatment maintains functional responses as well as tissue integrity both of which are observed to decline significantly with age in animal models of retinal diseases. Similar phenotypic pathology and age related degeneration trends are seen in human patients suffering from blinding diseases such as Leber congenital amaurosis (LCA) and retinitis pigmentosa (RP). Retinoid therapies therefore have the potential to improve the quality of life in patients suffering from genetic retinal diseases by delaying progressive vision loss.

## 2. Absorption and Distribution of Dietary Vitamin A as Retinyl Esters and Provitamin A Carotenoids

*De novo* synthesis of vitamin A is limited to plants and some microorganisms, therefore all vertebrates, including humans, must obtain vitamin A from dietary sources either as preformed vitamin A or provitamin A carotenoids [6]. The majority of vitamin A in the mammalian diet is not present in the free retinol form, but instead as both retinyl esters in animal tissues, and carotenoids contained in plant material. Preformed vitamin A, in the form of retinol or retinyl esters, is found almost exclusively in animal goods such as dairy products and organ meats such as liver [7,8]. Conversely provitamin A carotenoids, such as β-carotene, can be found in green leafy vegetables, such as spinach, as well as a variety of fruits such as apricots and papaya [9]. The intestinal mucosa is the active site for the uptake of free retinol, cleavage of carotenoids, and the hydrolysis of retinyl esters in vertebrates [10].

### 2.1. Intestinal Uptake and Metabolism of Pro-Vitamin A Carotenoids

Carotenoids are naturally occurring isoprenoid compounds (C40) produced by plants and some microorganisms. Carotenoids contain conjugated double bonds in the form of a polyene hydrocarbon chain, which is responsible for a variety of red and orange pigments that absorb light in the range of 300–600 nm [11]. Carotenoids in nature commonly contain a terminal benzene ring, which is either oxygenated or unoxygenated to yield molecules termed xanthophylls and carotenes respectively. Carotenoids serve many functions in mammalian biology including inherent antioxidant capabilities, incorporation into the macular region of the central human retina, and conversion into retinoid signaling molecules involved in development and gene regulation [12,13,14].

**Figure 1:**
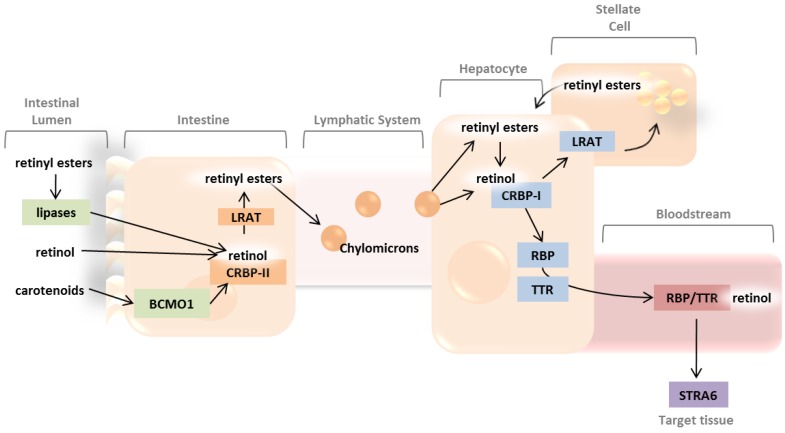
The metabolism of carotenoids and retinoids begin in the intestinal lumen, where provitamin A and retinoid molecules are absorbed. Retinyl esters are transported to the liver via the lymphatic system, while retinol is transported through the bloodstream before delivery to target tissues, such as the retina.

When ingested by mammalians carotenes are considered pro-vitamin A compounds since vertebrates have the ability to enzymatically transform various dietary carotenes into vitamin A. The first step in vitamin A metabolism begins with the symmetric cleavage of a carotene, such as β-carotene, by an enzyme at the intestinal brush boarder termed β,β-carotene 15,15′-monooxygenase (BCMO1) (Figure 1). BCMO1 cleaves carotenes at C15′/C15′ of the carbon backbone yielding two retinaldhyde molecules. BCMO1 converts a limited number of carotenoids to retinoid products *in vivo*, while a related protein BCDO2 (β,β-carotene 9,10-dioxygenase) cleaves carotenoids asymmetrically at the C9′/C10′ bond, and displays a broader substrate specificity [15]. Studies in BCMO1 knockout mice demonstrate that BCDO2 cannot compensate for the loss in carotene cleavage and vitamin A production, demonstrating the non-overlapping role of the two oxygenases [16].

Cell culture studies have suggested that the uptake of β-carotene, a common carotenoid in the human diet, is a saturable and regulated process controlled by the intestine-specific homeobox transcription factor (ISX) [17,18]. In a previous study, large doses of β-carotene were administered to healthy volunteers and less than half of the provitamin was reported to be converted to retinol, suggesting that the enzymatic cleavage of β-carotene to retinol is regulated in a dose dependent manner [19].

### 2.2. Intestinal Uptake of Retinyl Esters and Reesterification of Retinol by LRAT

Dietary retinoids are efficiently absorbed in the small intestine, but must be converted to the alcohol form of vitamin A before cellular transport. Dietary retinyl esters are hydrolyzed to retinol in the intestinal lumen or at the brush broader of enterocytes by pancreatic lipase or phospholipase B respectively [15,20]. Enterocyte specific uptake of free retinol or recently hydrolyzed retinyl ester is facilitated by cellular retinol binding protein II (CRBP-II) which binds the hydrophobic molecule with high affinity and transports it within the cytosol [21]. Three distinct retinol binding proteins exist in mammalians, CRBP-I is expressed ubiquitously in tissues while CRBP-II is both primarily and highly expressed in the jejuna mucosa suggesting its unique role in retinol absorption in the intestine, while CRBP-III primarily is found in heart, muscle, adipose and mammary tissue [22,23,24].

Following enterocyte uptake, free retinol is reesterified with long chain fatty acids, such as palmitate, and is secreted into the lymphatic system in the form of chylomicrons (Figure 1). Reesterification is accomplished by an acyltransferase enzyme, lecithin: retinol acyltransferase (LRAT), before incorporation into nascent chylomicrons [25]. Nascent chylomicrons include newly formed retinyl esters as well as other dietary lipids such as cholesterol, and enter the general circulation through the thoracic duct where they are further metabolized into smaller particles termed chylomicron remnants and distributed to the liver and other tissues [26]. The majority of absorbed free retinyl esters are also packaged into chylomicrons and secreted in the lymphatic system [27]. The remaining unabsorbed retinyl esters are systemically circulated and taken up by target tissues such as adipose, heart, muscle and lung tissue [28].

### 2.3. Systemic Circulation and Cellular Uptake by STRA6

Following hepatic uptake of retinyl esters hydrolysis of the ester linkage forms a retinol molecule, which binds immediately to the intercellular retinol-binding protein CRBP-I. A portion of retinol remains bound to intracellular CRBP-I, but the majority quickly becomes reesterified by LRAT, and stored within liver stellate cells [29]. Retinol stored as retinyl esters accumulate in the highest amounts in the liver but are also stored in tissues such as adipose, lung, and retinal pigment epithelium [30,31,32,33]. Secretion of retinol from these organs, with the exception of the RPE, into the systemic blood stream maintains normal blood retinol levels, even under times of diet insufficiency. Circulating plasma retinol is transported via a retinol-binding protein (RBP) and transthyretin (TTR) complex, and is required for transport since the retinol molecule alone is highly lipophilic (Figure 1). Genetic knockouts of *Rbp* in mice show that without the RBP transport protein these animals are exceedingly sensitive to vitamin A deficiency because of the inability to mobilize hepatic stores, and continue to have low serum retinol concentrations even after supplementing the diet [34]. In humans a deficiency of RBP results in a progressive atrophy of the retinal pigment epithelium and difficulty in dark adaptation, but patients are otherwise unaffected in other organs, perhaps due to the delivery of retinyl esters to tissues by chylomicron remnants [35]. TTR on the other hand may play a minor role in the transport of retinol since studies with TTR deficient mice show that mutants are healthy and fertile, despite extremely low retinol and circulating RBP levels [36]. However, the binding of TTR is believed to reduce the glomeruli filtration rate of RBP by increasing the molecular weight of the complex and therefore decreasing vitamin A urinary excretion [37].

Binding of the RBP-TTR-retinol complex to the plasma membrane receptor stimulated by retinoic acid gene 6 (STRA6) of a target cell releases the vitamin from its carrier and facilitates cellular uptake (Figure 1). STRA6 is highly expressed in cells or tissues, which depend on vitamin A for proper function. In the eye, retinal pigment epithelium cells highly express STRA6 near the basolateral membrane, allowing for efficient transport of vitamin A from the choroidal blood circulation, therefore allowing retinol to enter the visual cycle. Suppressing STRA6 expression in RPE cells has been observed to cause a decrease in the uptake of vitamin A in the eye, whereas up regulation of STRA6 by retinoic acid stimulation enhances vitamin A uptake [38]. Clinically, mutations in STRA6 cause various pathological phenotypes in humans including anophthalmia, mental retardation, congenital heart defects and embryonic lethality [39,40]. In the mouse retina specifically mutations in the *Stra6* gene lead to the development of short rod and cone photoreceptors, reduced scotopic and photopic ERG responses as well as optically dense vitreous humor [41].

## 3. Incorporation of Retinol into the Retina and Visual Cycle

The vertebrate retina contains both rod and cone photoreceptors, which are specialized for low intensity and high intensity light respectively. Rod photoreceptors are efficient single-photon detectors allowing for visual perception in low illumination. However, cone photoreceptors are far less sensitive but because of the varying sensitivities of opsin molecules these cells can distinguish various wavelengths of light allowing for the perception of color (reviewed in [42]).

Visual perception relies on the cyclic processing of 11-*cis*-retinal and its binding to a special class of light sensing GPCRs within photoreceptors cells, termed opsins to form visual pigments as rhodopsin or cone opsins. The light sensitive component of the human retina is comprised mainly of rod and cone photoreceptors cells, both, which utilize the 11-*cis*-retinal chromophore for visual transduction. The steady supply of 11-*cis*-retinal is maintained by cooperative enzymatic processing occurring between outer segments of both types of photoreceptor cells and the RPE layer, or between cone outer segments and Müller cells. Collectively these processes are referred to as the visual cycle. In many human retinal diseases these cyclic processes are disturbed resulting in an inability to either produce an adequate supply of 11-*cis*-retinal or a failure to remove the build-up of various retinoid products.

### 3.1. RPE and the Photoreceptor Visual Cycle

**Figure 2:**
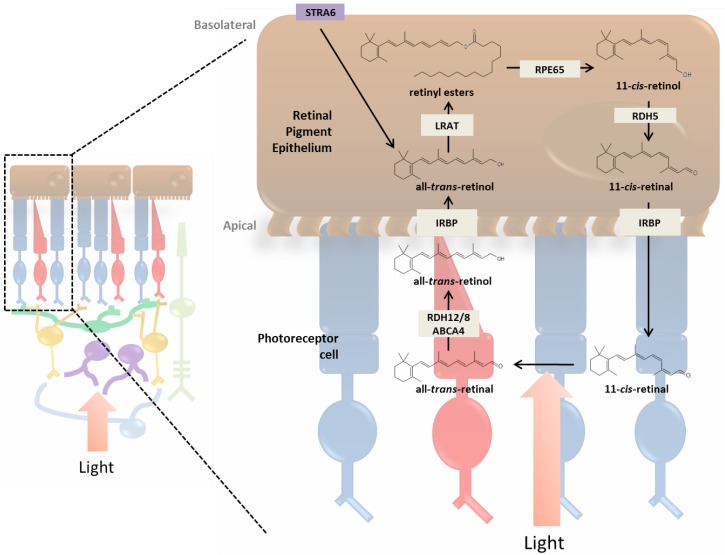
Visual cycle. Absorption of light by visual pigments (rhodopsin or cone opsin) causes isomerization of 11-*cis*-retinal to all-*trans*-retinal, resulting in phototransduction. Decay of activated rhodopsin yields opsin and all-*trans*-retinal, which is released and pumped out into the cytosol by a photoreceptor specific ATP-binding transporter (ABCA4) and reduced to all-*trans*-retinol by all-*trans*-retinal dehydrogenases (RDH8 and RDH12). All-*trans*-retinol diffuses into the RPE where it is esterified by lecithin:retinol acyltransferase (LRAT) to all-*trans*-retinyl esters, which are stored in retinosomes. All-*trans*-retinyl esters are isomerized to 11-*cis*-retinol in a reaction involving a 65 kDa RPE-specific protein (RPE65). To complete the visual cycle, 11-*cis*-retinol is then oxidized by 11-*cis*–retinal specific RDH (RDH5) to 11-*cis*-retinal, which then diffuses back into the photoreceptor where it combines with opsin to regenerate visual pigments. IRBP, interphotoreceptor retinoid-binding protein; Stra6, stimulated by retinoic acid gene 6.

The RPE contains a cascade of proteins required for the enzymatic isomerization of all-*trans*-retinol into the light sensitive chromophore 11-*cis*-retinal (Figure 2). All-*trans*-retinol is transported to RPE through the choridal blood circulation or photoreceptor outer segments. All-*trans*-retinol is subsequently absorbed on the basolateral side of the cell by the receptor STRA6 from the choridal blood circulation, which is facilitated by membrane bound LRAT. All-*trans*-retinol is transported though the interface of photoreceptor outer segments and microvilli of RPE via interphotoreceptor matrix and interphotoreceptor retinoid-binding protein (IRBP) (Figure 1, Figure 2). In both transport pathways, LRAT is necessary for the efficient uptake and usage of retinol in the RPE since it supplies esterified retinoid substrates for the formation of 11-*cis*-retinol via retinal pigment epithelium-specific 65 kDa protein (RPE65). In addition, retinyl esters not catalyzed by RPE65 accumulate to form retinyl esters used for retinoid storage in RPE specific organelles termed retinosomes [31,38,43,44]. Genetic knockout of the *Lrat* gene in mice has been observed to hinder vitamin A uptake in the gut, abolish the production of retinyl esters in most tissues (excluding adipose tissue which utilizes a different pathway for retinyl ester formation), and severely impair visual function [45,46]. In the retina a complete lack of retinyl ester formation results in the absence of 11-*cis*-retinal and therefore impairs the regeneration of rhodopsin, consequently this deficiency leads to progressive retinal degeneration manifested by the shortening of rod outer segments.

### 3.2. Müller Cells and the Cone Visual Cycle

Cone photoreceptor cells utilize a second pathway to regenerate chromophore independent of the RPE. The existence of this second cycle allows for rapid cone pigment regeneration under constant and bright illumination where pigment is rapidly bleached [47]. Unlike rod cells which solely rely on the RPE to convert all-*trans*-retinol to 11-*cis*-retinol, evidence from various species have suggested that Müller cells in the neural retina have the ability to perform this isomerization step autonomously from the RPE [48,49]. In addition, recent biochemical evidence has suggested that cones cells oxidize 11-*cis*-retinol to 11-*cis*-retinal thus allowing for faster chromophore recycling than seen in rod photoreceptors [42,50]. The cone specific visual cycle is evolutionarily conserved in numerous rod dominated and cone dominated species signifying the importance of this cycle despite the photoreceptor ratio variation seen between species [50].

### 3.3. Enzymatic Processing of Retinol in the RPE

The next step in the visual cycle after retinol esterification is the combined isomerization and hydrolysis of retinyl esters by the isomerohydrolase protein RPE65 [46,51,52]. This reaction yields 11-*cis*-retinol which further becomes oxidized by 11-*cis*-retinol dehydrogenase (RDH5) to 11-*cis*-retinal, additional dehydrogenases are also known to be involved in this oxidation including RDH11 and RDH10 (reviewed in [53]). Analogous to the *Lrat* deletion, genetic deletion or mutation of the *Rpe65* gene produces an intrinsic 11-*cis*-retinoid deficiency leading to the rapid onset of retinal degeneration and blindness [54]. Unlike *Rpe65* and *Lrat*, genetic knockout of *Rdh5* does not produce a drastic phenotype in mice except for an observed increase in *cis*-retinols and retinyl esters [43]. The absence of pathology observed in *Rhd5* knockout mice is most likely explained by the redundancy that exists within the retinol dehydrogenase family. Currently the view is that other proteins in the dehydrogenase family are exploited when RDH5 is insufficient [55]. The formation of 11-*cis*-retinal is the last enzymatic step in the visual cycle before the retinoid is transported through the interphotoreceptor matrix to the photoreceptor outer segment where it will bind to one of many opsin proteins and undergo light induced isomerization.

### 3.4. Retinoid Transport between RPE and Photoreceptor Cells

Interphotoreceptor retinoid-binding protein (IRBP) is the major soluble protein that exists in the interphotoreceptor matrix (IPM), and functions as the two-way carrier of retinoids, both from the RPE to photoreceptors and from photoreceptors back to RPE [56,57] (Figure 2). Surprisingly the rod visual cycle in *Irbp* knockout mice remains intact, although 11-*cis*-retinal regeneration occurs at a reduced rate when compared to identically reared WT mice [58]. Recently IRBP has been found to be crucial for the cone visual cycle most notably for proper cone function, maintenance of cone outer segments and eye development [58,59,60]. In *Xenopus* IRBP was found to bind specifically to the pericellular matrix of cone outer segments and Müller cell microvilli, suggesting that IRBP plays a significant role in the transport of retinoids to these cell types [61]. It is unknown whether other proteins are involved in this transport, and some potential candidates have been investigated, though the data are not conclusive [62]. Efficient transport is certainly necessary for the proper recycling of retinoids during the visual cycle because of their hydrophobicity, thus undiscovered secondary and compensatory transport mechanisms existing in the interphotoreceptor matrix may still remain to be uncovered.

### 3.5. Photoreceptor Cells and Visual Transduction

Once inside the photoreceptor cell the newly formed 11-*cis*-retinal forms a covalent schiff base bond with an opsin molecule contained within an outer segment disc membrane [63,64]. Incoming photons must pass through all layers of the retina before reaching photoreceptor outer segments and initiating phototransduction (Figure 2). Photon absorption by 11-*cis*-retinal changes the bound retinoid configuration from *cis* to *trans*, and allows the opsin molecule to activate the regulatory protein transducin through its own conformation change to MetaII. Transducin activation is accomplished by the catalytic exchange of GDP for GTP facilitated by photoactivated opsin. This exchange leads to a decrease of cytoplasmic cGMP concentrations, and eventually a nerve response is propagated to the brain and perceived as vision (phototransduction and visual processing reviewed in [65,66,67]). Rod and cones cells bind distinct transducin proteins, however by employing comparable genomics it was found that all forms of vertebrate opsin contain the same functional domains for binding transducin, confirming the importance of this signaling pathway in vision [68].

During transducin activation the schiff base bond between the opsin molecule and the newly isomerized all-*trans*-retinal is hydrolyzed. This hydrolysis forms free all-*trans*-retinal which subsequently becomes reduced to all-*trans*-retinol and binds to the cytosolic protein cellular retinol-binding protein type-1 (CRBP1) where it is transported out of the photoreceptor cell and back to the RPE for regeneration [69]. Excessive exposure to light, or a genetic mutation in one of the many essential visual cycle proteins can cause the accumulation of all-*trans*-retinal leading to the formation of condensation products, such as A2E, and cell toxicity [70,71,72].

## 4. Deficiencies in 11-*cis*-Retinal and Associated Retinal Degenerative Diseases

Mouse models of 11-*cis*-retinal deficiency have provided invaluable data regarding the importance of sustained retinoid cycling between the RPE and photoreceptor cells in vision; in addition, these models have provided biologically similar models of human retinal degeneration diseases for study. Both *Lrat* and *Rpe65* knockout mouse models have been employed in this field of research because of their inability to produce the essential chromophore 11-*cis*-retinal, and thus these animals develop retinal pathology similarly to what is observed in certain human retinal dystrophies.

### 4.1. Pathophysiology of 11-*cis*-Deficient Retinal Diseases in Mouse Models of Retinal Degeneration

The progressive loss of rod photoreceptors and shortening of rod outer segments with age has been reported in mice lacking functional *Lrat* or *Rpe65* [45,52]. Additionally both mouse models show rapid degeneration of cone photoreceptors, with complete degeneration of M/L and S opsin occurring at P28 and P42 respectively [73,74]. Both knockout mice display mislocalization of cone opsin at P28 and upon repeated administration of 11-*cis*-retinal opsin trafficking can be partially corrected in young *Lrat^−/−^* and *Rpe65^−/−^* mice, emphasizing the importance of 11-*cis*-retinal for proper cone opsin conformation and trafficking [54,74,75]. Recently, mounting evidence supports the hypothesis that mislocalization of cone opsin results in increased endoplasmic reticulum stress and induces the early cone cell death seen in LCA mouse models. Pharmacological studies in *Lrat^−/−^* and *Rpe65^−/−^* mice have demonstrated that cone cell death can be ameliorated by both the ER chemical chaperone tauroursodeoxycholic acid, and proteasome inhibitor MG-132 respectively, suggesting a central role for ER in opsin protein degradation [76,77].

Rod opsin on the other hand is observed to traffic normally in the absence of 11-*cis*-retinal, both in *Lrat* and *Rpe65* knockout mice, suggesting that rod pigment does not necessarily need its chromophore for proper photoreceptor localization [74], whereas 11-*cis*-retinal deficiency can induce abnormality of length and morphological structures of rod outer segments in LCA mouse models [74,78]. Experiments in P23H mutant mice have shown that the 11-*cis*-retinal or 9-*cis*-retinal chromophore is important for increasing protein stability and intracellular transportation of mutant rod opsin, providing evidence that specific protein sequences may also be important for proper functioning and transport of rod opsin [79,80,81].

**Figure 3:**
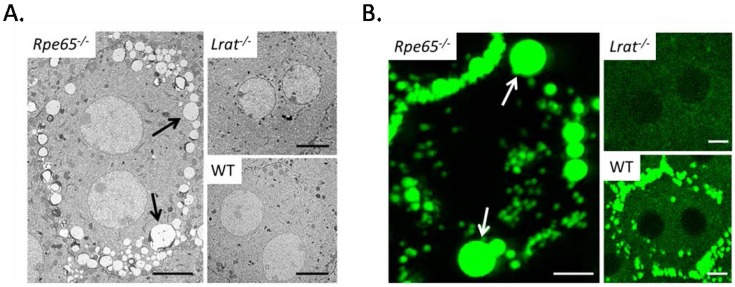
(**A**) Horizontal EM images of RPE in 3 month old *Rpe65^−/−^*, *Lrat^−/−^* and wild-type mice. Of particular interest are the large retinosomes present around the perimeter of RPE cells in *Rpe65^−/−^* mice (black arrows), these formations are indicative of excessive ester accumulation in the retina. (**B**) Two photon imaging of the RPE in 3 month old *Rpe65^−/−^*, *Lrat^−/−^* and wild-type mice. Large autofluorescent spots are observed in the RPE of *Rpe65^−/−^* mice (white arrows), while such spots are absent in *Lrat^−/−^* mice, and are minimally observed in wild type mice. Scale bar 5.0 µm.

The cyclic processing of chromophore can be blocked by removal or mutation in any one of the enzymes required for regeneration. Furthermore cycle interruption may result not only in cessation of 11-*cis*-retinal chromophore production but also in the accumulation of products from the previous steps, leading to cell disruption and death. Mutations in *Lrat* and *Rpe65* disrupt the visual cycle at distinct steps in regeneration and therefore knockout animals present differences in retinoid composition in the eye. The loss of functional RPE65 prevents the conversion of stored retinyl esters to 11-*cis*-retinal and causes the unrestrained accumulation of retinyl esters in the RPE, leading to a nonfunctioning visual cycle [52]. Excessive ester accumulation, appearing as retinosomes, can be clearly seen in young *Rpe65^−/−^* mice, while such structures are rare in either *Lrat^−/−^* or wild-type mice (Figure 3). In addition noninvasive two-photon imaging techniques have revealed analogous fluorescent structures in RPE of 3 months old *Rpe65^−/−^* mice, while these structures were completely absent from *Lrat^−/−^*, and minimal in wild-type mice [44].

Whereas *Rpe65^−/−^* mice exhibit extremely high concentrations of retinyl ester, mice lacking the enzyme LRAT possess only trace amounts of retinyl esters in the RPE, but likewise have a no detectable 11-*cis*-retinal in the retina, indicating that LRAT is essential for the esterification step of the visual cycle [74]. Furthermore, LRAT activity is required for storage of retinyl esters in other tissues, such as the liver and lungs, thus in addition to the retinal degenerative phenotype *Lrat^−/−^* mice are highly susceptible to vitamin A deficiency [30,82]. In conclusion, because of the pathological similarities shared between select human retinal degenerative diseases and both *Rpe65^−/−^* and *Lrat^−/−^* mice these models provide practical avenues for comprehensively studying aspects of human disease progression and potential treatments.

4.2. 11-*cis*-Retinal Deficiency and Leber Congenital Amaurosis

Defects in 11-*cis*-retinal regeneration are seen in a number of human inherited degenerative retinopathies including early childhood onset Leber congenital amaurosis (LCA). Diagnosis of LCA is usually confirmed early in life by irregular electroretinographic and papillary responses, and vision commonly declines with age until complete blindness is observed by the third or fourth decade of life [83,84]. Numerous gene mutations have been reported to cause LCA in humans including RPE65, LRAT, CRX (Homeodomain transcription factor), CRB1 (Crumbs like protein 1), TULP1 (Tubby-like protein), AIPL1 (aryl hydrocarbon interacting protein), and various other genes [84]. LCA typically is an autosomal recessive inherited disease, though autosomal dominant patterns have been reported [85]. The early-onset rod-cone dystrophy phenotype is observed in human patients diagnosed with LCA, but also in *Rpe65^−/−^* and *Lrat^−/−^* mice, as discussed above [45,52,86]. Early in life patients with LCA exhibit visual impairment with attenuated rod and cone function, macular atrophy, severely delayed or minimal ERG responses, nystagmus, and retinal cell degeneration [83,84,87,88]. LCA is currently considered an incurable disease but several promising therapies are presently being investigated, including gene therapy and chromophore replacement therapy [89,90,91].

## 5. Artificial Visual Chromophore Therapeutics and Further Applications

**Figure 4:**
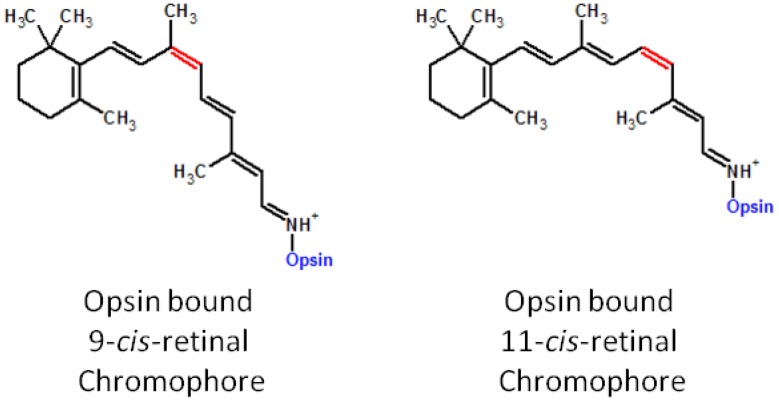
Isomers of the opsin chromophore. Both 11-*cis*-retinal and 9-*cis*-retinal form a Schiff base with residue K296 of the opsin molecule forming the light sensitive chromophore used in vision.

Pharmacological replacement of 11-*cis*-retinal has been shown to be highly effective at reconstituting functional visual pigment, increasing ERG responses and reducing the rate of retinal degeneration in animals with *Rpe65* or *Lrat* mutations [89,91]. 9-*cis* isomers have proven to be the most successful isomer in replacing the native 11-*cis* chromophore *in vivo*. Moreover 9-*cis*-retinal is preferred over 11-*cis*-retinal for chromophore replacement therapy because of its increased stability, ease of synthesis and ability to form light sensitive isorhodopsin *in vivo* [91]. Once incorporated into the rod outer segment 9-*cis*-retinal forms a schiff base with residue K296 of opsin producing a chromophore molecule analogous to 11-*cis*-retinal bound chromophore, and reducing the amount of endogenous opsin apoprotein [92] (Figure 4).

9-*cis-*retinoids taken orally, are converted to pro-drug forms *in vivo*, stored in the liver, transported in the blood, and eventually taken up into retinal tissue similar to dietary vitamin A. The capability to store 9-*cis* retinoids in tissues that naturally sequester vitamin A is especially important in producing a continuous therapeutic effect with retinoid drug administration. To avoid potential negative effects of administering large doses of retinoids future development of targeted delivery systems may lead to lower toxicity and improved effectiveness [93,94].

### 5.1. Prevention of the LCA Phenotype with Administration of 9-*cis*-Retinoids in Animal Models

**Figure 5:**
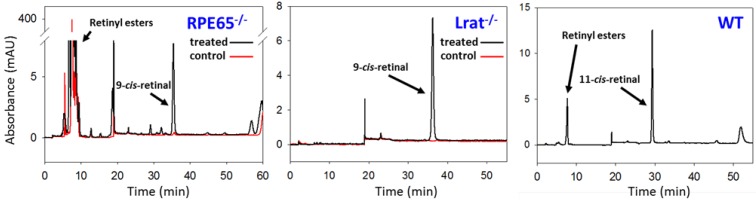
Normal phase HPLC analysis of retinoids in dark adapted mouse models of retinal degeneration. Concentrations of *cis*-retinoids and retinyl esters in the retina differ before and after 9-*cis*-retinoid treatment. Control *Rpe65* knockout mice exhibit significantly increased concentrations of retinyl esters and are devoid of 11-*cis*-retinal. *Rpe65^−/−^* mice treated with 9-*cis*-retinoids show increase 9-*cis*-retinal bound to rhodopsin. Likewise, untreated *Lrat* knockout mice lack both 11-*cis*-retinal and retinyl esters, while treated *Lrat* knockout mice show an increase 9-*cis*-retinal bound to rhodopsin. WT mice show normal concentrations of 11-*cis-*retinal, as well as small amounts of retinyl esters.

The *Lrat^−/−^* and *Rpe65^−/−^* mouse provide useful disease models for studying the efficacy and toxicity of chromophore replacement therapy, since these mice lack visual chromophore and develop retinopathy that closely resembles LCA in humans. In *Rpe65^−/−^* mice oral gavage of 9-*cis*-retinal, the biologically active form, which binds with opsin, has been shown to produce functional isorhodopsin, restore rod function, increase light sensitivity, and reduce RPE ester concentration *in vivo* [89,91]. Likewise, intravitreal injections of 9-*cis*-retinal increased ERG responses and improved obstacle avoidance in a RPE65 deficient canine model of LCA [95]. 9-*cis*-retinal treatment in both *Lrat^−/−^* and *Rpe65^−/−^* mice demonstrate the ability of this retinal isomer to bypass essential steps in retinoid regeneration, fully integrate into photoreceptor outer segments and form isorhodopsin capable of sensing light (Figure 5).

Various analogs of 9-*cis*-retinal have been investigated that theoretically provide better stability in gastric acidity when ingested and are further metabolized in the liver to produce storage forms of 9-*cis-*retinyl esters, such as 9-*cis-*retinyl palmitate [93,96]. Prolonged improvements in ERG responses in 9-*cis* retinyl acetate treated *Rpe65^−/−^* mice implies that 9-*cis*-retinoids are stored in the liver, mobilized, taken up by RPE cells through the circulation as 9-*cis*-retinol and incorporated into the visual cycle similar to the all-*trans* isomer [97]. Uptake of retinol from the circulation into the RPE of *Rpe65^−/−^* mice remains functional despite the presence of an abnormally large quantity of retinyl esters, implying that circulating 9-*cis*-retinoids can be absorbed by the RPE even though a functional visual cycle does not exist [98]. Prodrugs, which can be stored by the body, provide a practical approach to 9-*cis*-retinal delivery in humans by increasing drug bioavailability and decreasing the need for frequent dosing.

9-*cis*-retinyl acetate is one such prodrug, since it must be metabolized to the active 9-*cis-*retinal by the liver and then delivered to the eye via the bloodstream. Analogous to 9-*cis*-retinal therapy 9-*cis*-retinyl acetate treatment in *Lrat^−/−^* and *Rpe65^−/−^* mice generates functional isorhodopsin, maintains retinal thickness, and attenuates the decrease in ERG scotopic and photopic responses consistently seen with increasing age [71,99]. Similar to rod photoreceptors, the rate of age related cone photoreceptor cell death was slowed in 9-*cis*-retinyl acetate treated *Rpe65^−/−^* and *Lrat^−/−^* mice compared to untreated counterparts [100,101]. Furthermore significantly improved pure cone cell ERG responses were recorded in 9-*cis*-retinyl acetate treated *Rpe65^−/−^* and *Lrat^−/−^* mice lacking the functional transducin protein required for continued phototransduction in only rod cells. These data suggest that chromophore replacement therapy may be beneficial for also rescuing cone photoreceptor cells in LCA patients, which tend to be lost earlier in disease progression than rod photoreceptor cells [100]. Pharmacokinetic and systemic retinal toxicity studies demonstrated that WT, Rpe65*^−/−^*, and *Lrat^−/−^* mice administered various dosing regimens of 9-*cis*-retinyl acetate displayed no toxicity at therapeutic dosing [97,99]. A recent study demonstrated that retinas of Rpe65*^−/−^* and *Lrat^−/−^* mice were well tolerated to continuous exposure of high levels of QLT091001, a 9-*cis*-retinyl acetate drug, without the accumulation of toxic retinoid byproducts, such as A2E, and obvious pathological changes in neural retina and RPE [99]. Importantly QLT091001, developed by QLT, Inc., has been tested in preliminary human clinical trials (ClinicalTrials.gov number, NCT01014052).

More recently, subcutaneous implantations of microparticle-hydrogels loaded with 9-*cis*-retinyl acetate have improved ERG responses and maintained retinal morphology in *Lrat^−/−^* mice, suggesting that the use of such implants can reduce the frequency of dosing and therefore decrease the risk of hypervitaminosis A in patients [94,102,103,104,105,106,107]. The side effects observed from extreme over supplementation of vitamin A derivatives have prompted extensive research and development of chemically modified retinoids, as well as novel delivery systems that decrease toxicity and increase drug effectiveness. Excess natural or synthetic retinoids pose serious teratogenic risks and can possibly lead to craniofacial, cardiac, thymic, and central nervous system malformations in infants [106,107,108]. In adults chronic hypervitaminosis A, caused by long-term retinoid administration, can result in fibrosis and cirrhosis of the liver, hypercalcemia and bone loss [102,103,104]. The severe side effects observed from over supplementing vitamin A derivatives have recently prompted extensive research and development of chemically modified retinoids, as well as novel delivery systems that decrease toxicity and increase drug effectiveness. Advancements in slow release therapies may in the future reduce the necessity for frequent dosing allowing patients to visit clinicians less frequently, while providing a consistent dosing of the drug.

### 5.2. Therapeutics of 9-*cis*-Carotenoids in the Treatment of LCA

Proretinoid compounds, such as carotenoids, have also been investigated for their potential use in treating chromophore deficiency in retinal diseases. Of particular interest is the naturally occurring carotenoid isomer 9-*cis*-β-carotene, because if metabolized and cleaved symmetrically by BCMO1, this carotene isomer has the potential to produce 9-*cis*-retinal *in vivo*. In addition substituting carotenes in place of retinoid supplements is particularly appealing since it eliminates the risk of developing hypervitaminosis A, given that β-carotene uptake and catabolic cleavage is negatively regulated by dietary vitamin A intake [17,109].

Recently a clinical study in patients with fundus albipunctatus, a congenital form of night blindness resulting from a genetic mutation in gene RDH5 required for the oxidation of 11-*cis*-retinol to 11-*cis*-retinal, demonstrated that administration of 9-*cis*-carotene rich supplements improved ERG responses and enhanced patients mean visual field score [110,111]. Conversely, a similar experiment was performed in both the *Lrat^−/−^* and *Rpe65^−/−^* mouse and demonstrated extremely limited delivery of 9-*cis*-retinal to the eye when these animals were administrated 9-*cis*-β-carotene isolated from *D. barawil* extracts [112]. Furthermore it was demonstrated *in vitro* that a second carotenoid cleavage enzyme, β-carotene dioxygenase 2 (BCDO2), exists in the intestine and favorably cleaves 9-*cis*-β-carotene asymmetrically, producing products which are further metabolized into all-*trans*-retinal by BCMO1 [112]. The results from the latter study provide evidence that *cis*-carotenoids are far less effective than 9-*cis*-retinoids for delivery of 9-*cis*-retinal to the eye because of the different catabolic pathways used to produce active retinoid compounds from proretinoids. The above mentioned clinical study included a small sample size of seven patients, and did not contain a control or placebo group; therefore the results observed in this study could be attributed to the increase in the overall intake of dietary vitamin A and not the specific action of the 9-*cis* isomer of β-carotene.

## 6. Conclusions

To sustain vision vertebrates require the continued cyclic regeneration of the vitamin A derivative 11-*cis*-retinal. Prolonged insufficient dietary supply of vitamin A, select genetic defects in genes required for the production of 11-*cis*-retinal chromophore or discontinuous retinoid cycling may have devastating effects on the overall health of the retina and the quality of vision. Particular human diseases, such as LCA, lack the functional enzymatic reactions to regenerate 11-*cis*-retinal, and therefore patients with these defects exhibit decreased visual responses at an early age, which additionally decline steadily throughout life.

Supplementation with preformed *cis*-retinoid derivatives has been show to bypass defective steps in the visual cycle, and regenerate pigments necessary for vision in animal models of retinal degenerative diseases. The main caveats to retinoid treatment are the myriad of toxic effects seen with administration of pharmacological doses of vitamin A derivatives for prolonged lengths of time. Therefore novel methods for reducing the risk of vitamin A toxicity, improving drug effectiveness, and reducing the frequency of dosing are absolutely necessary for designing a safe retinoid derived drug for treatment of retinal degenerative diseases associated with chromophore deficiency.
